# Supplementary material for: Polarization Control via Artificial Optical Nonlinearity in Dielectric Metasurfaces
Source: ACS Nano. 2026 Jan 21;20(4):3499–509. doi: 10.1021/acsnano.5c13948 (PMC12874632; doi:10.1021/acsnano.5c13948)
Supplement: Supplementary file 1 [file nn5c13948_si_001.docx]

**Supporting Information: Polarization control via artificial optical nonlinearity in dielectric metasurfaces**

*Fuyong Yue^1, †,§^, Giacomo Balistreri^1,†^, Nicola Montaut^1^, Fabrizio Riminucci^2,3^, Andrea Toma^4^, Riccardo Piccoli^1,#^, Stefano Cabrini^3^, Roberto Morandotti^1^*, Luca Razzari^1^**

^1^Institut National de la Recherche Scientifique, Centre Énergie Matériaux Télécommunications (INRS-EMT), 1650 Boulevard Lionel-Boulet, Varennes, Québec J3X 1P7, Canada

^2^Dipartimento di Fisica, Università del Salento, Strada Provinciale Lecce-Monteroni, Campus Ecotekne, Lecce 73100, Italy

^3^Molecular Foundry, Lawrence Berkeley National Laboratory, One Cyclotron Road, Berkeley, California 94720, USA

^4^Istituto Italiano di Tecnologia, Via Morego 30, Genova 16163, Italy

E-mail: [roberto.morandotti@inrs.ca](mailto:roberto.morandotti@inrs.ca)

E-mail: [luca.razzari@inrs.ca](mailto:luca.razzari@inrs.ca)

^†^ These authors contributed equally to this work.

^§^ Present address: School of Microelectronics, University of Science and Technology of China, Hefei, Anhui 230026, China

^#^ Present address: Department of Molecular Sciences and Nanosystems, Ca’ Foscari University of Venice, via Torino 155, 30172, Venice, Italy.

**Section 1**. **Third-harmonic generation in cuboid-shaped dielectric meta-atoms.**

The response of an optical material to an applied light field, $\tilde{E}(\omega)$, is described by the polarization density (i.e., the dipole moment per unit volume), $\tilde{P}$. The nonlinear component of the polarization density for a third-order process, $\tilde{P}^{NL}$,can be written as:^1,2^

$\tilde{P}^{NL}=\left[ \begin{matrix} P_{x}^{NL} \\ P_{y}^{NL} \\ P_{z}^{NL} \end{matrix} \right]=\varepsilon_{0}M\left[ \begin{aligned} \begin{matrix} E_{x}^{3}(\omega) \\ E_{y}^{3}(\omega) \end{matrix} \\ E_{z}^{3}(\omega) \\ 3E_{y}(\omega)E_{z}^{2}(\omega) \\ 3E_{z}(\omega)E_{y}^{2}(\omega) \\ 3E_{x}(\omega)E_{z}^{2}(\omega) \\ 3E_{z}(\omega)E_{x}^{2}(\omega) \\ 3E_{x}(\omega)E_{y}^{2}(\omega) \\ 3E_{y}(\omega)E_{x}^{2}(\omega) \\ 6E_{x}(\omega)E_{y}(\omega)E_{z}(\omega) \end{aligned} \right]$ (S1)

where$M=\left( \begin{matrix} \begin{matrix} \chi_{11} \\ \chi_{21} \\ \chi_{31} \end{matrix} & \begin{matrix} \chi_{12} \\ \chi_{22} \\ \chi_{32} \end{matrix} \end{matrix} \begin{matrix} \begin{matrix} \chi_{13} \\ \chi_{23} \\ \chi_{33} \end{matrix} & \begin{matrix} \chi_{14} \\ \chi_{24} \\ \chi_{34} \end{matrix} \end{matrix} \begin{matrix} \begin{matrix} \chi_{15} \\ \chi_{25} \\ \chi_{35} \end{matrix} & \begin{matrix} \chi_{16} \\ \chi_{26} \\ \chi_{36} \end{matrix} & \begin{matrix} \chi_{17} \\ \chi_{27} \\ \chi_{37} \end{matrix} \end{matrix} \begin{matrix} \begin{matrix} \chi_{18} \\ \chi_{28} \\ \chi_{38} \end{matrix} & \begin{matrix} \chi_{19} \\ \chi_{29} \\ \chi_{39} \end{matrix} & \begin{matrix} \chi_{1,10} \\ \chi_{2,10} \\ \chi_{3,10} \end{matrix} \end{matrix} \right)$ is the third-order nonlinear susceptibility matrix. Here the compact form $\chi_{lm}$ is used to represent the elements of the fourth-rank tensor, where *l* is 1, 2, 3, corresponding to the *x*, *y*, *z* components of the nonlinear polarization density, respectively, and *m* is 1, 2, …, 10, corresponding to the combinations of polarization components of the fundamental light given by *xxx*, *yyy*, *zzz*, *yzz*, *yyz*, *xzz*, *xxz*, *xyy*, *xxy*, *xyz*, respectively. Using the symmetry conditions outlined in Ref. ^2^, the analysis of the nonlinear response of materials can be simplified.


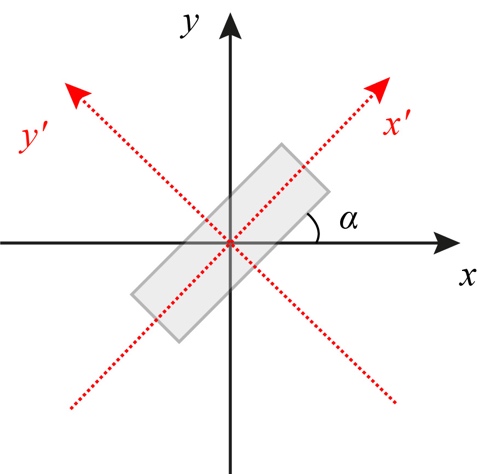


**Figure S1**. Schematic of a rotated cuboid under the normal (*x*, *y*) and local ($x^{'}, y'$) coordinates, where $\alpha$ is the rotation angle.

We consider the general case where an arbitrary-polarized fundamental light field $\tilde{E}(\omega)=\frac{1}{2}\tilde{A}e^{-i(\omega t-kz)}+c.c$ propagating along *z* with frequency $\omega$ and complex amplitude $\tilde{A}=A_{0}\left[ \begin{matrix} cos\theta\\ e^{i\delta}sin\theta\end{matrix} \right]$ (where $\theta$ is the polarization angle, and $\delta=\phi_{x}-\phi_{y}$ is the phase difference between the *x*- and *y*- polarization components; note that $\tilde{A}$ represents the Jones vector associated with $\tilde{E}$) interacts with rotated cuboids with rotation angle $\alpha$ (see Fig. S1). The polarization characteristics of the third-harmonic (TH) light are derived by using the circular polarization basis.^3^ The analysis is divided into three steps. First, the polarization state of the fundamental light is projected from the normal coordinates ($x$, $y$) into the local coordinates ($x'$, $y'$), with the $x'$ ($y'$) axis coinciding with the long (short) axis of the rotated cuboids (see Fig. S1). The Jones vector of the input light, $\tilde{A}'$, under the local coordinates can be obtained by multiplying the Jones vector of the original input light, $\tilde{A}$, with the rotation matrix $J\left( \alpha\right)=\left[ \begin{matrix} cos\alpha& -sin\alpha\\ sin\alpha& cos\alpha\end{matrix} \right]$. For ease of calculation, we define the input polarization using the circular polarization basis as $\tilde{A}=A_{0}\left[ \begin{matrix} cos\theta\\ e^{i\delta}sin\theta\end{matrix} \right]=\psi_{R}|\left. R \right\rangle+\psi_{L}|\left. L \right\rangle$, where $|\left. R \right\rangle$ and $|\left. L \right\rangle$ are the normalized right- and left-handed circular polarization states, $|\left. R \right\rangle=\frac{1}{\sqrt{2}}\left[ \begin{matrix} 1 \\ -i \end{matrix} \right]$ and $|\left. L \right\rangle=\frac{1}{\sqrt{2}}\left[ \begin{matrix} 1 \\ i \end{matrix} \right]$, and $\psi_{R}$ and $\psi_{L}$ are the corresponding amplitudes given by $\psi_{R}=\frac{A_{0}}{\sqrt{2}}(cos\theta+ie^{i\delta}sin\theta)$ and $\psi_{L}=\frac{A_{0}}{\sqrt{2}}(cos\theta-ie^{i\delta}sin\theta)$, respectively. Therefore, the new Jones vector under the local coordinates can be expressed as

$\tilde{A}^{'}=J\left( \alpha\right)\cdot\tilde{A}=\frac{1}{\sqrt{2}}\left[ \begin{matrix} \psi_{R}e^{i\alpha}+\psi_{L}e^{-i\alpha} \\ -i\psi_{R}e^{i\alpha}+i\psi_{L}e^{-i\alpha} \end{matrix} \right]$ (S2)

In the second step, the polarization density $\tilde{P}'$ is calculated under the local coordinates by employing the standard nonlinear optical equations for the third-harmonic generation (THG) process.^1,2^ As described in the main text (see Fig. 1(a)), a cuboid meta-atom has three mirror planes (m) and three 2-fold rotation axes (A2) that are perpendicular to these mirror planes. Therefore, the Hermann-Mauguin symbol is $\frac{2}{m}\frac{2}{m}\frac{2}{m}$ (otherwise known as *mmm*), which corresponds to the orthorhombic crystal class. The third-order susceptibility matrix of this class is $M=\left( \begin{matrix} \begin{matrix} \chi_{11} \\ 0 \\ 0 \end{matrix} & \begin{matrix} 0 \\ \chi_{22} \\ 0 \end{matrix} \end{matrix} \begin{matrix} \begin{matrix} 0 \\ 0 \\ \chi_{33} \end{matrix} & \begin{matrix} 0 \\ \chi_{24} \\ 0 \end{matrix} \end{matrix} \begin{matrix} \begin{matrix} 0 \\ 0 \\ \chi_{35} \end{matrix} & \begin{matrix} \chi_{16} \\ 0 \\ 0 \end{matrix} & \begin{matrix} 0 \\ 0 \\ \chi_{37} \end{matrix} \end{matrix} \begin{matrix} \begin{matrix} \chi_{18} \\ 0 \\ 0 \end{matrix} & \begin{matrix} 0 \\ \chi_{29} \\ 0 \end{matrix} & \begin{matrix} 0 \\ 0 \\ 0 \end{matrix} \end{matrix} \right)$.^1^ We consider the case when the incident light is polarized along the *x*-*y* plane and propagates in the *z* direction (i.e., $E_{z}=0$), and the field experiences negligible resonance effects within the meta-atoms. In this case, the THG polarization density can be simplified as:

$\tilde{P}^{'}\left( 3\omega\right)=\left[ \begin{matrix} {P^{'}}_{x}\left( 3\omega\right) \\ {P^{'}}_{y}\left( 3\omega\right) \end{matrix} \right]=\frac{1}{8}\varepsilon_{0}\left[ \begin{matrix} \begin{matrix} \chi_{11} & 0 \\ 0 & \chi_{22} \end{matrix} & \begin{matrix} \chi_{18} & 0 \\ 0 & \chi_{29} \end{matrix} \end{matrix} \right]\left[ \begin{matrix} \begin{matrix} {A'}_{x}^{3}\left( \omega\right) \\ {A'}_{y}^{3}\left( \omega\right) \end{matrix} \\ \begin{matrix} 3{A^{'}}_{x}\left( \omega\right){A'}_{y}^{2}\left( \omega\right) \\ 3A_{y}^{'}\left( \omega\right){A'}_{x}^{2}\left( \omega\right) \end{matrix} \end{matrix} \right]e^{-i3(\omega t-kz)}+c.c=\tilde{P}_{3\omega}^{'}e^{-i3(\omega t-kz)}+c.c$ (S3)

By substituting the polarization components from Eq. (S2) into Eq. (S3), we can write the nonlinear polarization density amplitude $\tilde{P}_{3\omega}^{'}$ as:

$\tilde{P}_{3\omega}^{'}={a_{1}{(\psi}_{R}}^{3}e^{3i\alpha}|\left. L \right\rangle+{\psi_{L}}^{3}e^{-3i\alpha}|\left. R \right\rangle)+{a_{2}{(\psi}_{R}}^{3}e^{3i\alpha}|\left. R \right\rangle+{\psi_{L}}^{3}e^{-3i\alpha}|\left. L \right\rangle)+{a_{3}{(\psi}_{R}}^{2}{\psi_{L}e}^{i\alpha}|\left. L \right\rangle+{\psi_{R}\psi_{L}}^{2}e^{-i\alpha}|\left. R \right\rangle)+{a_{4}{(\psi}_{R}}^{2}{\psi_{L}e}^{i\alpha}|\left. R \right\rangle+{\psi_{R}\psi_{L}}^{2}e^{-i\alpha}|\left. L \right\rangle)$ (S4)

where $\left\{ \begin{matrix} \begin{matrix} a_{1}=\varepsilon_{0}\frac{\chi_{11}-3\chi_{18}+\chi_{22}-3\chi_{29}}{32} \\ a_{2}=\varepsilon_{0}\frac{\chi_{11}-3\chi_{18}-\chi_{22}+3\chi_{29}}{32} \end{matrix} \\ \begin{matrix} a_{3}=\varepsilon_{0}\frac{{3\chi}_{11}+3\chi_{18}-3\chi_{22}-3\chi_{29}}{32} \\ a_{4}=\varepsilon_{0}\frac{{3\chi}_{11}+3\chi_{18}+3\chi_{22}+3\chi_{29}}{32} \end{matrix} \end{matrix} \right.$

In the final step, the nonlinear polarization density amplitude is transformed back to the normal coordinates ($x$, $y$) by calculating the product of $\tilde{P}_{3\omega}^{'}$ and the rotation matrix $J\left( -\alpha\right)=\left[ \begin{matrix} cos\alpha& sin\alpha\\ -sin\alpha& cos\alpha\end{matrix} \right]$. The final polarization density amplitude $\tilde{P}_{3\omega}$ under the normal coordinates can be expressed as

$\tilde{P}_{3\omega}=J\left( -\alpha\right){{*\tilde{P}_{3\omega}^{'} =a}_{1}{(\psi}_{R}}^{3}e^{4i\alpha}|\left. L \right\rangle+{\psi_{L}}^{3}e^{-4i\alpha}|\left. R \right\rangle)+{a_{2}{(\psi}_{R}}^{3}e^{2i\alpha}|\left. R \right\rangle+{\psi_{L}}^{3}e^{-2i\alpha}|\left. L \right\rangle)+{a_{3}{(\psi}_{R}}^{2}{\psi_{L}e}^{2i\alpha}|\left. L \right\rangle+{\psi_{R}\psi_{L}}^{2}e^{-2i\alpha}|\left. R \right\rangle)+{a_{4}{(\psi}_{R}}^{2}\psi_{L}|\left. R \right\rangle+{\psi_{R}\psi_{L}}^{2}|\left. L \right\rangle)$(S5)

This expression allows us to fully characterize the polarization and phase of the resulting TH light for any arbitrary polarization of the fundamental light.

**Section 2. Simulated electric field components within a cuboid meta-atom at the wavelength of 1596 nm.**


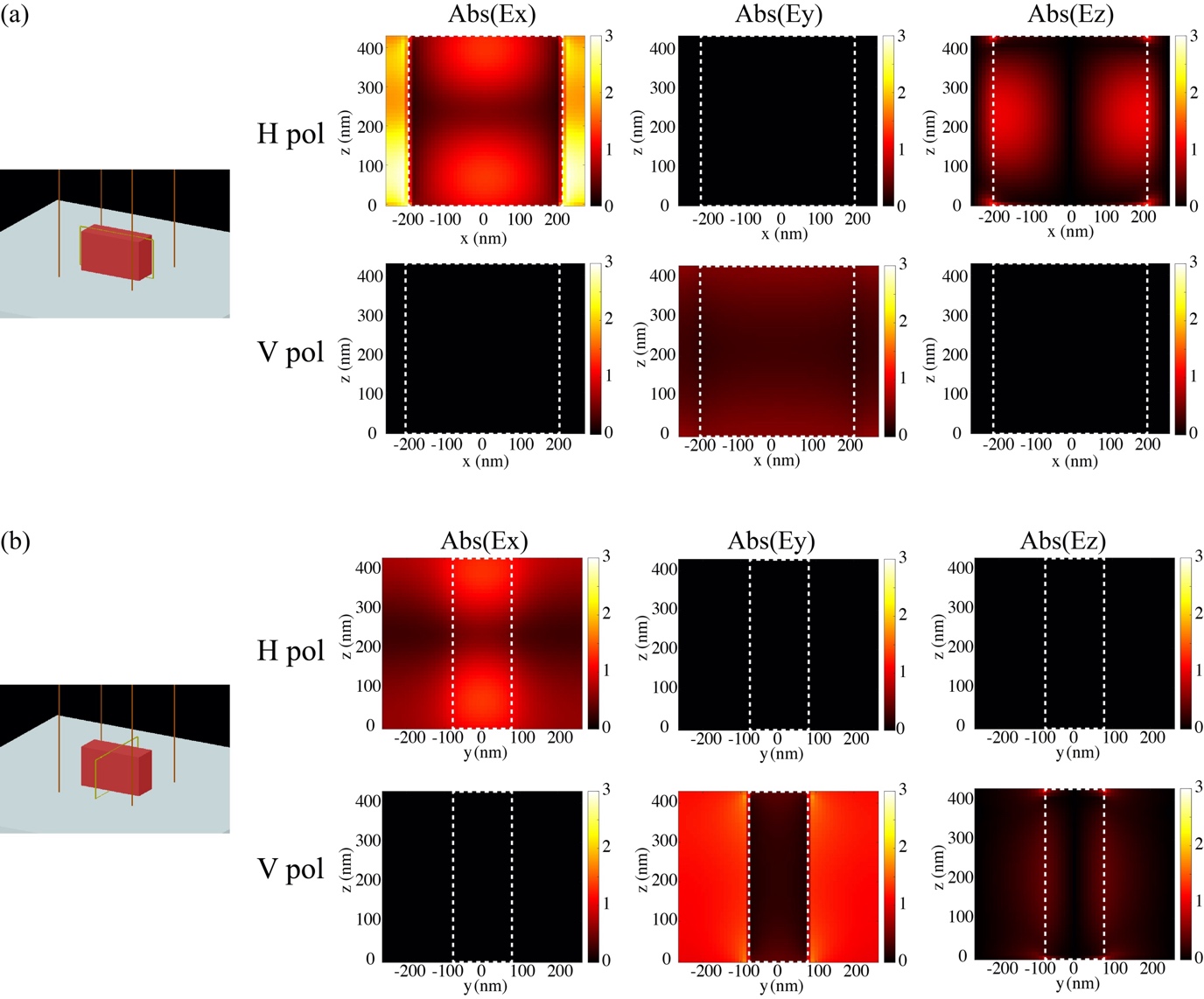


**Figure S2**. The simulated amplitudes of the electric field components abs(Ex), abs(Ey), abs(Ez) at the two cross-sections indicated by the yellow frames in the cuboid meta-atom diagrams (left images in (a) and (b)) for an H (i.e., along the meta-atom long axis, top row) and V (i.e., perpendicular to the meta-atom long axis, bottom row) input polarized plane wave at a wavelength of $\lambda_{f}$ = 1596 nm. The meta-atom boundaries are outlined by white dashed lines in the electric field simulation panels.

**Section 3. Experimental setup.**


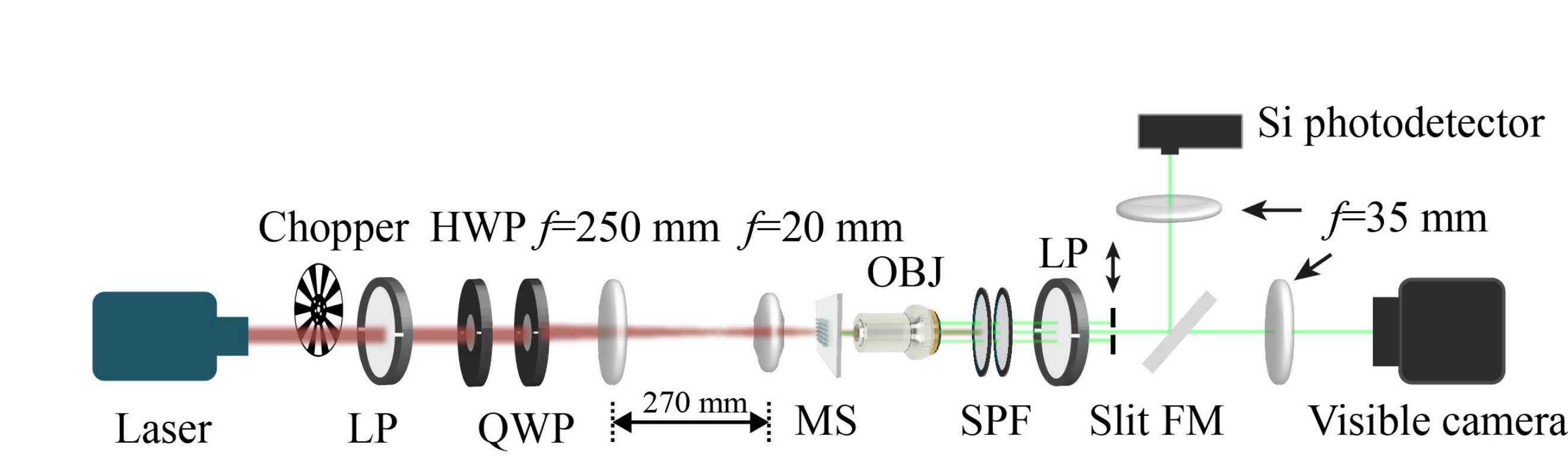


**Figure S3**. Schematic of the experimental setup. LP: linear polarizer. HWP: half-wave plate. QWP: quarter-wave plate. MS: metasurface. OBJ: objective lens with NA of 0.4. SPF: short-pass filters. FM: flip mirror. The quarter-wave plate and/or the output linear polarizer were inserted or removed from the setup according to the measurement requirements.

**Section 4. THG spectral and spatial characterization of the plain metasurface.**

The THG spectral characterization of the plain metasurface was performed by detecting the TH light via a visible spectrometer. Figure S4 shows the measured normalized THG spectra for fundamental wavelengths in the range of $\lambda_{f}=$1516 nm – 1676 nm, with a wavelength step of $\Delta\lambda_{f}=20$ nm (the input polarization was fixed to H, representing the most efficient polarization for THG, see main text). The spectral dependency of the TH light with respect to the fundamental wavelength was found to follow the expected trend, i.e., $\lambda_{THG}=\frac{\lambda_{f}}{3}.$


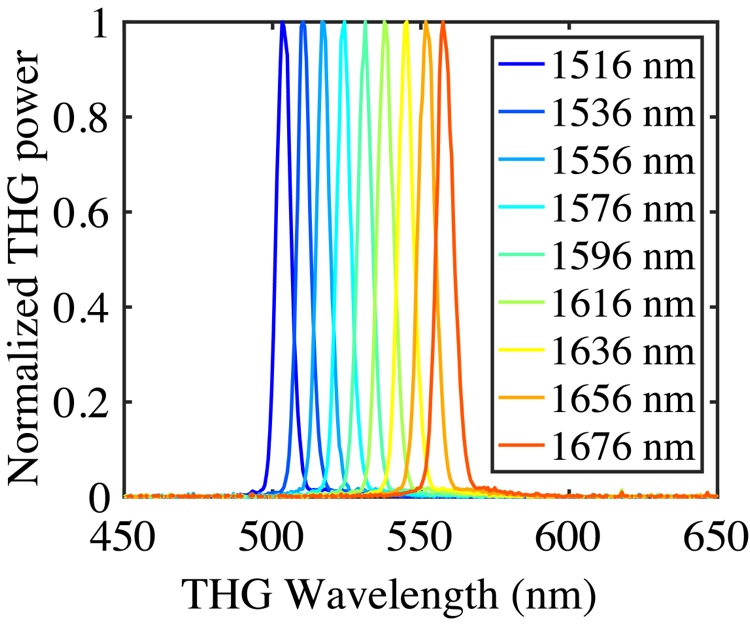


**Figure S4**. Normalized THG spectra obtained by tuning the fundamental wavelength.

Using a visible camera, we also mapped the TH spatial intensity profiles generated from the plain metasurface (and compared it with the ones of a uniform a-Si film of same thickness) when the input polarization was set to H, V, LCP, and RCP. The captured TH intensities are depicted in Figure S5. As can be seen, only zeroth-order TH beams were detected from the plain metasurface. Due to the isotropic nature of the homogeneous a-Si film, its response to H or V polarizations was practically identical.^1^ The isotropic film further showed a strong suppression of THG under circularly polarized fundamental inputs. In contrast, the plain metasurface showed a stronger response for H fundamental polarization compared to V polarization, as well as significant THG with circularly polarized input light. For more details about this comparison, see main text.


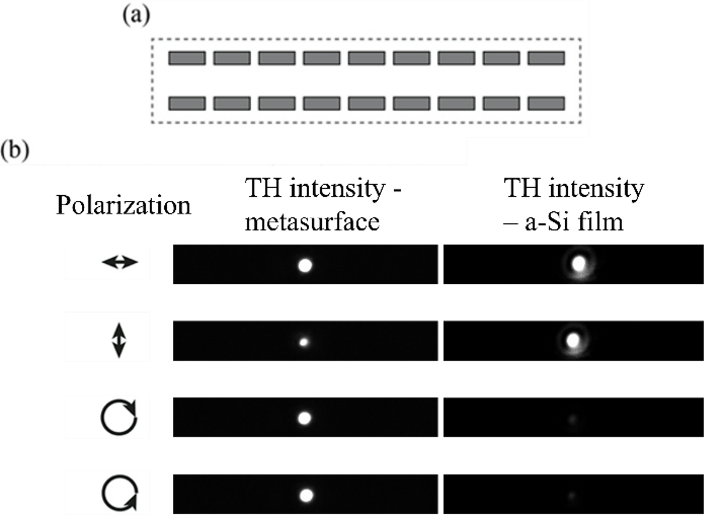


**Figure S5**. (a) Sketch of the plain metasurface. (b) Captured spatial intensity profiles of the TH light emitted by the plain metasurface (left column) and by a film of the same material and thickness (right column). The images are taken using a visible camera, when the unrotated meta-atoms and film were exposed to different input fundamental polarizations (i.e., H, V, RCP, and LCP).

**Section 5. Retrieving the nonlinear tensor elements from the THG efficiency.**

The coupled wave equations for the THG process can be retrieved following an approach similar to that used for second harmonic generation.^1,2^ We start from the scalar nonlinear wave equation in one dimension:

$\frac{\partial^{2}E_{i}}{\partial z^{2}}+\frac{\omega_{i}^{2}}{c^{2}}n^{2}(\omega_{i})E_{i}=-\frac{\omega_{i}^{2}}{\varepsilon_{0}c^{2}}P_{i}^{NL}$ (S6)

where $E_{i}={\frac{1}{2}(A}_{\omega_{i}}e^{-i\left( \omega_{i}t-k_{i}z \right)})+c.c$ is the electric field at the frequency $\omega_{i}$, $A_{\omega_{i}}$ is the field amplitude, $k_{i}$ and $n(\omega_{i})$ are the corresponding wave-vector and refractive index, $c$ is the speed of light, and $P_{i}^{NL}$ is the nonlinear polarization density term. The wave equation must be valid for all $\omega_{i}$ frequency components involved in the process. For the case under study, we have the following:

$E_{\omega}=\frac{1}{2}\left( A_{\omega}e^{-i\left( \omega t-k_{\omega}z \right)} \right)+c.c$ (S7)

$E_{3\omega}=\frac{1}{2}(A_{3\omega}e^{-i\left( 3\omega t-k_{3\omega}z \right)})+ c.c$ (S8)

$P\left( 3\omega\right)={\frac{1}{8}\varepsilon}_{0}\chi^{\left( 3 \right)}A_{\omega}^{3}e^{-i3\left( \omega t-k_{\omega}z \right)}+c.c{=P}_{3\omega}e^{-i3(\omega t-k_{\omega}z)}+c.c$ (S9)

where $k_{\omega}=\frac{n_{\omega}\omega}{c}$ and $k_{3\omega}=\frac{n_{3\omega}3\omega}{c}$ are the wave-vectors, while $n_{\omega}$ and $n_{3\omega}$ ($n_{\omega}^{2}= \varepsilon_{\omega}$, $n_{3\omega}^{2}=\varepsilon_{3\omega}$) are the material refractive indices at the fundamental and TH frequencies, respectively. By substituting Equations (S7-S9) into Equation (S6), we can obtain the following:^2^

$\left[ \frac{d^{2}A_{3\omega}}{dz^{2}}+2ik_{3\omega}\frac{dA_{3\omega}}{dz}-k_{3\omega}^{2}A_{3\omega}+\frac{\varepsilon_{\omega}\left( 3\omega\right)^{2}A_{3\omega}}{c^{2}} \right]e^{-i({3\omega t- k}_{3\omega}z)}+c.c=-\frac{1}{8}\frac{\chi^{\left( 3 \right)}\left( 3\omega\right)^{2}}{c^{2}}A_{\omega}^{3}e^{-i3\left( {\omega t- k}_{\omega}z \right)}+c.c$ (S10)

We can drop the complex conjugate terms from each side of Equation (S10) and still maintain the equality and we can cancel the factor $e^{-i3\omega t}$ on each side. Moreover, we can assume the slowly-varying amplitude approximation, i.e., $\left| \frac{d^{2}A_{3\omega}}{dz} \right|\ll\left| k_{3\omega}\frac{dA_{3\omega}}{dz} \right|$, so that we obtain the first of the equations in the coupled wave model:^2^

$\frac{dA_{3\omega}}{dz}=\frac{3i\omega}{8n_{3\omega}c}\chi^{\left( 3 \right)}A_{\omega}^{3}e^{i\Delta kz}$ (S11)

where $\Delta k=3k_{\omega}-k_{3\omega}$ is the wavevector mismatch. In a similar manner, we can retrieve the equation for the fundamental field amplitude, $A_{\omega}$, thus obtaining the following system of equations:

$\left\{ \begin{aligned} \frac{dA_{\omega}}{dz}=\frac{3i\omega}{8n_{\omega}c}\chi^{(3)}A_{3\omega}{A_{\omega}^{*}}^{2}e^{-i\Delta kz} \\ \frac{dA_{3\omega}}{dz}=\frac{3i\omega}{8n_{3\omega}c}\chi^{(3)}{A_{\omega}}^{3}e^{i\Delta kz} \end{aligned} \right.$ (S12)

Since only a negligible fraction of the pump power is transferred to the TH light, we can use the undepleted pump approximation and consider $A_{\omega}$ as a constant. Hence, the electric field amplitude of the TH light, $A_{3\omega}$, can be written as:

$$A_{3\omega}\left( l \right)=\int_{0}^{l} \frac{dA_{3\omega}}{dz}dz=\frac{3i\omega}{8n_{3\omega}c}\chi^{\left( 3 \right)}{A_{\omega}}^{3}\int_{0}^{l} e^{i\Delta kz}dz$$

$=\frac{3i\omega}{8n_{3\omega}c}\chi^{(3)}{A_{\omega}}^{3}l{\cdot e}^{i\Delta kl/2}\cdot sinc \left( \Delta kl/2 \right)$ (S13)

where *l* is the length of the material along the propagation direction, *z*.

The relation between the intensity and amplitude of a plane wave is:

$I=\frac{1}{2}\varepsilon_{0}nc\left| A \right|^{2}$ (S14)

where $\varepsilon_{0}$ is the vacuum permittivity.

Thus, the intensity of TH light can be expressed as:

$I_{3\omega}= \frac{1}{2}\varepsilon_{0}n_{3\omega}c\left| A_{3\omega} \right|^{2}=\left[ \frac{9\pi^{2}{\chi^{\left( 3 \right)}}^{2}l^{2}}{{4\varepsilon}_{0}^{2}n_{3\omega}n_{\omega}^{3}c^{2}\lambda_{f}^{2}}\cdot{sinc}^{2} \left( \frac{\Delta kl}{2} \right) \right]I_{\omega}^{3}$ (S15)

where $\lambda_{f}$ is the wavelength corresponding to the fundamental field. The intensity-to-intensity THG conversion efficiency $\eta_{I}$ can be written as:

$\eta_{I}=\frac{I_{3\omega}}{I_{\omega}}=\left[ \frac{9\pi^{2}{\chi^{\left( 3 \right)}}^{2}l^{2}}{{4\varepsilon}_{0}^{2}n_{3\omega}n_{\omega}^{3}c^{2}\lambda_{f}^{2}}\cdot{sinc}^{2} \left( \frac{\Delta kl}{2} \right) \right]I_{\omega}^{2}$ (S16)

The TH beam waist and pulse duration are $\sqrt{3}$ times smaller than those of the fundamental pulse, due to the cubic dependence between the corresponding intensities. For this reason, a factor of $3\sqrt{3}$ appears in the relation between the average power-to-power and intensity-to-intensity conversion efficiencies:

$\eta_{P}=\frac{\eta_{I}}{3\sqrt{3}}$ (S17)

The third-order susceptibility can thus be related to the power-to-power conversion efficiency:^4^

${|\chi}^{(3)}|= G\sqrt{\eta_{p}}$(S18)

where $G=\frac{2\varepsilon_{0}c\lambda_{f}\sqrt{n_{3\omega}n_{\omega}^{3}}}{\sqrt[4]{3}\pi lI_{\omega}sinc\left( \frac{\Delta kl}{2} \right)}$. Mathematically, the values of the third-order susceptibility tensor elements $\chi_{lm}$ can be estimated by measuring the conversion efficiency under specific input/output polarization settings. The THG polarization densities corresponding to $\chi_{11}$, $\chi_{22}$, $\chi_{18}$, $\chi_{29}$ can be rewritten as

$$P_{x}\left( 3\omega\right)=\frac{1}{8}\varepsilon_{0}\chi_{11}A_{x}^{3}\left( \omega\right)e^{-i3(\omega t-kz)}+c.c (S19)$$

$$P_{y}\left( 3\omega\right)=\frac{1}{8}\varepsilon_{0}\chi_{22}A_{y}^{3}\left( \omega\right)e^{-i3(\omega t-kz)}+c.c (S20)$$

$$P_{x}\left( 3\omega\right)=\frac{3}{8}\varepsilon_{0}\chi_{18}A_{x}(\omega)A_{y}^{2}\left( \omega\right)e^{-i3\left( \omega t-kz \right)}+c.c (S21)$$

$$P_{y}\left( 3\omega\right)=\frac{3}{8}\varepsilon_{0}\chi_{29}A_{y}(\omega)A_{x}^{2}\left( \omega\right)e^{-i3\left( \omega t-kz \right)}+c.c (S22)$$

$\chi_{11}$($\chi_{22}$) relates to the response of the polarization density $\tilde{P}(3\omega)$ in the *x* (*y*) direction, due to a field applied in the *x* (*y*) direction, and $\chi_{18}$ ($\chi_{29}$) relates to the response of the polarization density $\tilde{P}(3\omega)$ in the *x* (*y*) direction due to a field applied both in the *x* and *y* directions. Therefore, the tensor elements $\chi_{11}$, $\chi_{22}$, $\chi_{18}$, $\chi_{29}$, can be estimated based on Eq. (S18) and Eqs. (S19-S22) by measuring the power-to-power conversion efficiency from the plain metasurface ($\alpha=0$). In our evaluation of the artificial nonlinear response of the metasurface, we assume the effective values of the tensor elements to be positive. To quantify the values of such tensor elements, we first made three measurements with the same average fundamental power: 1) we set the input polarization to H and measured the THG conversion efficiency $\eta_{P1}$; 2) we set the input polarization to V and measured the THG conversion efficiency $\eta_{P2}$; 3) we set the input polarization to RCP and measured the THG conversion efficiencies for H ($\eta_{P3}$) and V ($\eta_{P4}$) polarization settings of the output linear polarizer. The relation between the tensor elements and these conversion efficiencies can be expressed as

$\left\{ \begin{matrix} \begin{matrix} \chi_{11}= G\sqrt{\eta_{P1}} \\ \chi_{22}= G\sqrt{\eta_{P2}} \end{matrix} \\ \begin{matrix} \left| \frac{\chi_{11}-3\chi_{18}}{2\sqrt{2}} \right|=G\sqrt{\eta_{P3}} \\ \left| \frac{\chi_{22}-3\chi_{29}}{2\sqrt{2}} \right|=G\sqrt{\eta_{P4}} \end{matrix} \end{matrix} \right.$ (S23)

The measured TH powers were $P_{1}=112.9\pm6.8 pW$, $P_{2}=1.0\pm0.3 pW$, $P_{3}=9.8\pm1 pW$, and $P_{4}=2.6\pm0.42 pW$, respectively, for the four conditions described above, when the input was set to $P_{in}=5\pm0.05 mW$. The corresponding power-to-power conversion efficiencies were found to be $\eta_{P1}=2.3\pm0.14\times{10}^{-8}$, $\eta_{P2}=2.0\pm1.0\times{10}^{-10}$, $\eta_{P3}=2.0\pm0.2\times{10}^{-9}$, and $\eta_{P4}=5.2\pm1.0\times{10}^{-10}$, respectively. The refractive index values of silicon at the fundamental and TH wavelengths ($n_{\omega}=3.48$ and $n_{3\omega}=4.42$) were used in this evaluation. The thickness of the metasurface layer was set to *l* = 425 nm. The pump peak intensity was $I_{\omega}=0.42\pm0.004 GW/cm^{2}$. Therefore the retrieved tensor elements were: $\chi_{11}=7.90\pm0.26\times{10}^{-18}\left( \frac{m^{2}}{V^{2}} \right)$, $\chi_{22}=0.74\pm0.12\times{10}^{-18}\left( \frac{m^{2}}{V^{2}} \right)$, $\chi_{29}=1.38\pm0.11\times{10}^{-18}\left( \frac{m^{2}}{V^{2}} \right)$, and $\chi_{18}=0.47\pm0.14\times{10}^{-18}\left( \frac{m^{2}}{V^{2}} \right)$ or $4.80\pm0.14\times{10}^{-18} \left( \frac{m^{2}}{V^{2}} \right)$. To identify the correct value of $\chi_{18}$ and further test our model, we measured the emitted TH power as a function of the input polarization angles (Fig. S6). The solid black and blue curves represent the estimated TH powers using Eq. (5) and Eq. (S18), by inserting the two possible sets of nonlinear tensor elements ($\chi_{11}, \chi_{22},\chi_{18}, \chi_{29}$), while the black stars are the measured data. As one can see, we were able to obtain a good fit to the measured trend by using the estimated $\chi_{18}=4.67\times{10}^{-19} \left( \frac{m^{2}}{V^{2}} \right)$. The close match between the measured and theoretical data further supports the accuracy of our derived model.

**
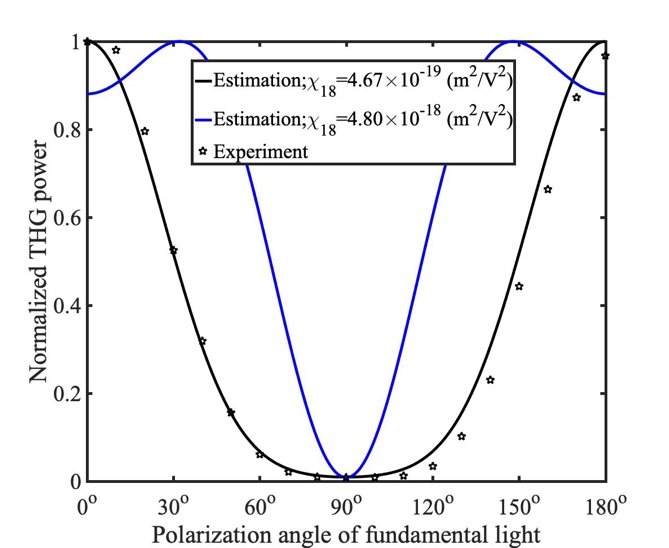
**

**Figure S6**. Estimated (black solid curve for $\chi_{18}$ = $4.67\times{10}^{-19} {(m}^{2}/V^{2})$, blue solid curve for $\chi_{18}$ = $4.80\times{10}^{-18} {(m}^{2}/V^{2})$) and measured (star markers) THG power as a function of the linear polarization angle of the input fundamental light.

**Section 6. Experimental characterization of the nonlinear polarization metagrating: additional results.**

We illuminated the nonlinear polarization metagrating with horizontally polarized fundamental light at $\lambda_{f}=$1596 nm and measured the THG diffraction pattern using a visible camera (Infinity2, Lumenera). The inset of Fig. S7(a) shows the diffraction pattern generated at $\lambda_{THG}=$532 nm (see spectra in Fig. S7(a)). A slit mounted on a translation stage was inserted into the output optical path to select the different diffraction orders for power quantification. The power of the $\pm$1^st^ orders of the TH light was measured using a silicon-based photodetector coupled to a lock-in amplifier and exhibited a cubic dependence with respect to the pump peak intensity (see Fig. S7(b)). Figure S7(c) shows the diffraction patterns of the TH light when different fundamental polarizations were used for illumination.

**

**

**Figure S7**. (a) Normalized THG spectra of the 0^th^ order and the $\pm1$^st^ orders of the generated TH light from the fabricated metagrating when horizontally polarized fundamental light was used (at $\lambda_{f}=$1596 nm). Inset: THG diffraction pattern captured using a visible camera. (b) Measured THG power of the $\pm1$^st^ orders as a function of pump peak intensity at $\lambda_{f}=$1596 nm. Solid line: cubic fit. Square and circle markers indicate the measured data of the -1^st^ order and the +1^st^ order, respectively. (c) Diffraction patterns of TH light for different polarizations of the fundamental light.

**Section 7. Nonlinear gradient metasurface: additional results.**

Figure S8 shows the diffraction patterns of the TH light generated by the nonlinear phase gradient metasurface when different input circular polarization states are used to excite it. A quarter wave plate and a linear polarization analyzer were placed before the detector to determine the polarization state of each diffraction order. These results are complementary to those shown in Figure 4 of the main manuscript. In particular, we can see that only positive (negative) diffraction orders are excited when using RCP (LCP) input light. Moreover, the ±1^st^ diffraction orders keep the same handedness of the fundamental light, whereas the ±2^nd^ diffraction orders exhibit an opposite handedness. These results are consistent with the model predictions.

~~
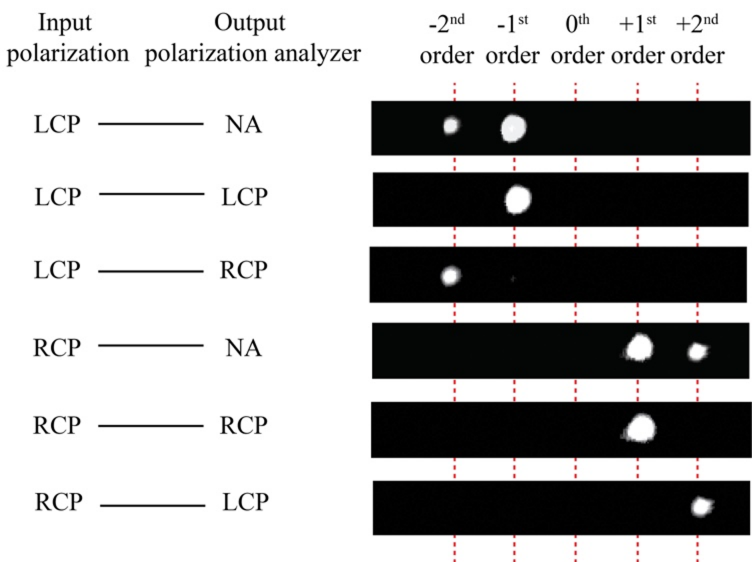
~~

**Figure S8**. Characterization of the circular polarization states of the diffraction orders from the nonlinear gradient metasurface under illumination of RCP and LCP fundamental light (NA stands for No Analyzer).

**References**

(1) Yang, X.-l.; Xie, S.-w. Expression of third-order effective nonlinear susceptibility for third-harmonic generation in crystals. *Appl. Opt.* **1995**, *34* (27), 6130-6135.

(2) Boyd, R. W. *Nonlinear optics*; Elsevier, 2003.

(3) Li, G.; Chen, S.; Pholchai, N.; Reineke, B.; Wong, P. W. H.; Pun, E. Y. B.; Cheah, K. W.; Zentgraf, T.; Zhang, S. Continuous control of the nonlinearity phase for harmonic generations. *Nat. Mater.* **2015**, *14* (6), 607-612.

(4) Yue, F.; Piccoli, R.; Shalaginov, M. Y.; Gu, T.; Richardson, K. A.; Morandotti, R.; Hu, J.; Razzari, L. Nonlinear Mid‐Infrared Metasurface based on a Phase‐Change Material. *Laser Photonics Rev.* **2021**, 2000373.
